# Supplementary material for: OAF is a DAF-like gene that controls ovule development in plants
Source: Commun Biol. 2023 May 8;6:498. doi: 10.1038/s42003-023-04864-5 (PMC10167350; doi:10.1038/s42003-023-04864-5)
Supplement: Supplementary file 2 — Supplementary Information [file 42003_2023_4864_MOESM2_ESM.pdf]

## Supplementary Fig. 1

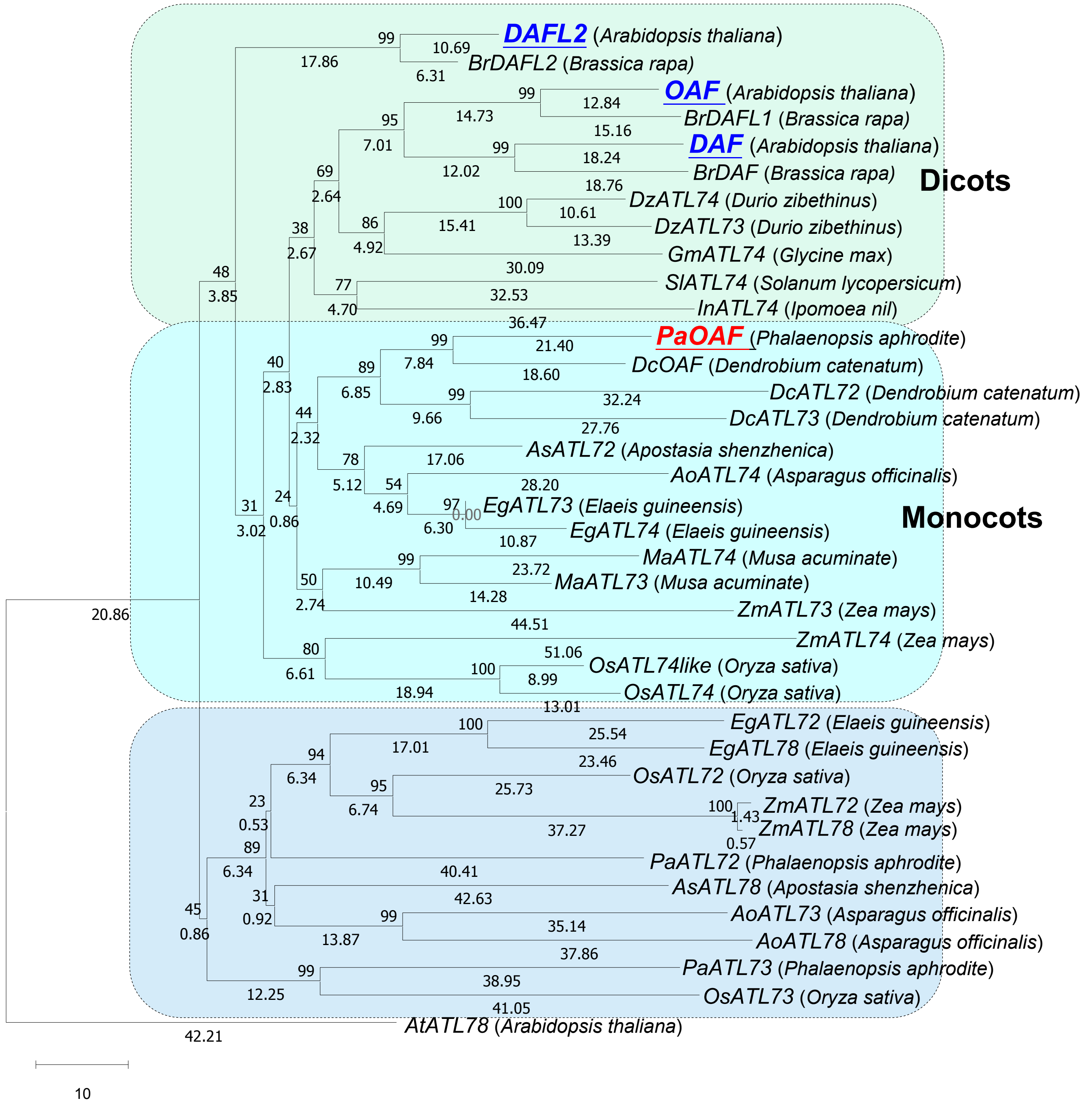

**Supplementary Fig. 1 Phylogenetic analysis of plant DAF/OAF-like proteins.**

Amino acid sequences of the plant DAF/OAF-like proteins were obtained via the National Center for Biotechnology Information server (http://www.ncbi.nlm.nih.gov/). Based on the protein sequence, the *Arabidopsis DAF* and *OAF* (in blue color) are closely related to each other and were assigned to the dicots group of *DAF/OAF*-like genes, which are separated from *Phalaenopsis PaOAF* (in red color) in the monocot group of *DAF/OAF*-like genes. *Arabidopsis DAFL2* (in blue color) was separated relatively far from *DAF/OAF* and *PaOAF.* Names of the plant species are listed behind each of the DAF/OAF-like protein names. The multiple sequence alignment was performed by using ClustalW of DNA Data Bank of Japan (DDBJ) (http://clustalw.ddbj.nig.ac.jp/top-e.html). The phylogenetic graph was generated by using the TREEVIEW program. Numbers on major branches indicate bootstrap percentages for 1,000 replicate analyses.

## Supplementary Fig. 2

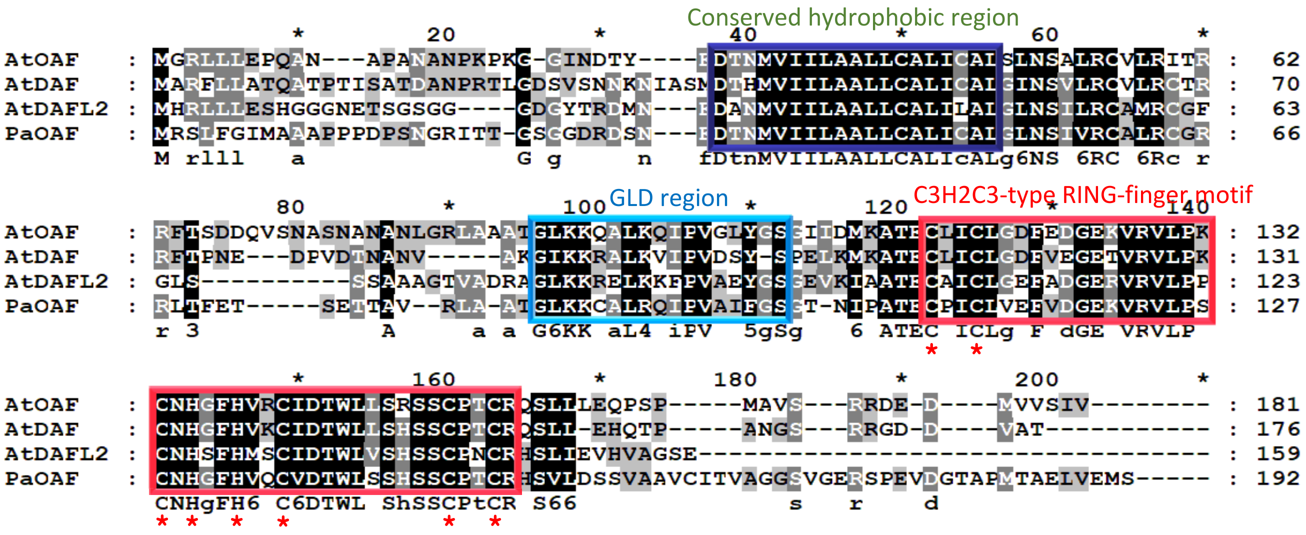


**Supplementary Fig. 2 Amino acid sequences alignment of three *Arabidopsis* and one *Phalaenopsis* DAF/OAF-like proteins.**

Alignment of the amino acid sequences for *Arabidopsis* OAF (AtOAF), DAF (AtDAF), DAFL2 (AtDAFL2) and *Phalaenopsis* PaOAF proteins. The conserved hydrophobic region was boxed in dark blue, the conserved GLD region was boxed in blue and the conserved C3H2C3-type RING-finger motif was boxed in red. The red star indicated the eight conserved C3H2C3 residues in the RING finger motif. The dark highlights indicate identical residues and gray highlights indicate similar residues. Dashes were introduced to improve alignment. The amino acid sequences were aligned by the BioEdit program using ClustalW Multiple Alignment. This sequence alignment was generated by the ClustalW-Multiple Sequence Alignment Program at the DNA Data Bank of Japan (http://clustalw.ddbj.nig.ac.jp/top-e.html).

## Supplementary Fig. 3

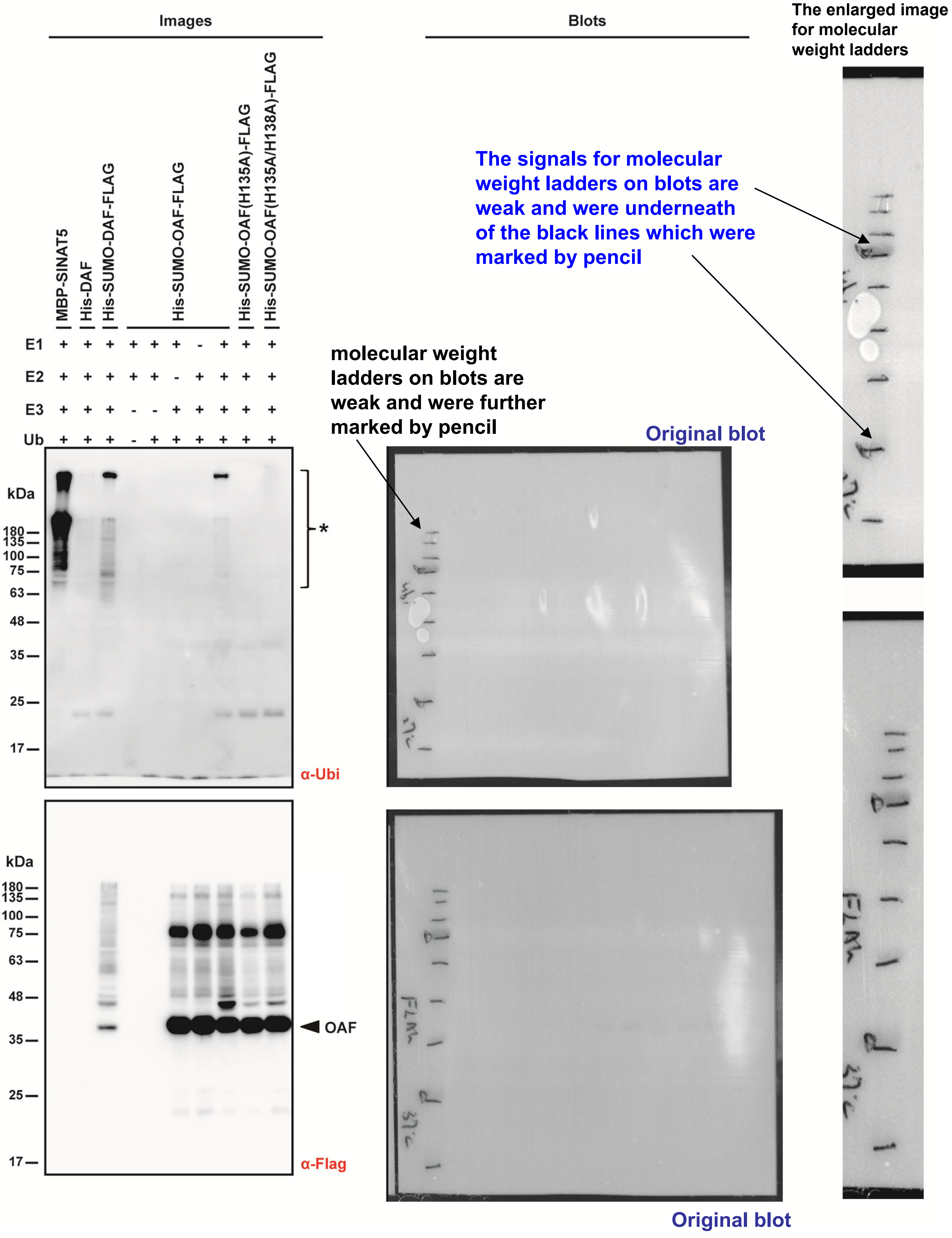

**Supplementary Fig. 3** Auto-ubiquitination activity of OAF and its RING mutants. His-SUMO-OAF-FLAG was assayed for E3 activity in the presence or absence of E1 (UBA1), E2 (UbcH5b) and Ubiquitin (Ub) (lanes 4-8). Poly-ubiquitinated OAF (Ub(n)-OAF) (lane 8) was detected with a polyclonal antibody against ubiquitin (top blot). His-SUMO-OAF-FLAG was detected by Western blotting using anti-Flag antibody (lanes 6-10) (bottom blot). MBP-SINAT5 (lane 1) and His-SUMO-DAF-FLAG (lane 3) were used as a positive control. No ubiquitinated signals were observed in the presence of OAF mutants His-SUMO-OAF(H135A)-FLAG (lane 9) and His-SUMO-OAF(H135A/H138A)-FLAG (lane 10). Asterisk indicate the signals of Ub(n)-OAF, Ub(n)-SINAT5 or Ub(n)-DAF. In this experiment, two unique blots were used and both blots shown in this Figure are uncropped. The signals for molecular weight ladders on blots are weak (indicated by arrows) and were underneath of the black lines which were marked by pencil in order to capture clear images.

## Supplementary Fig. 4

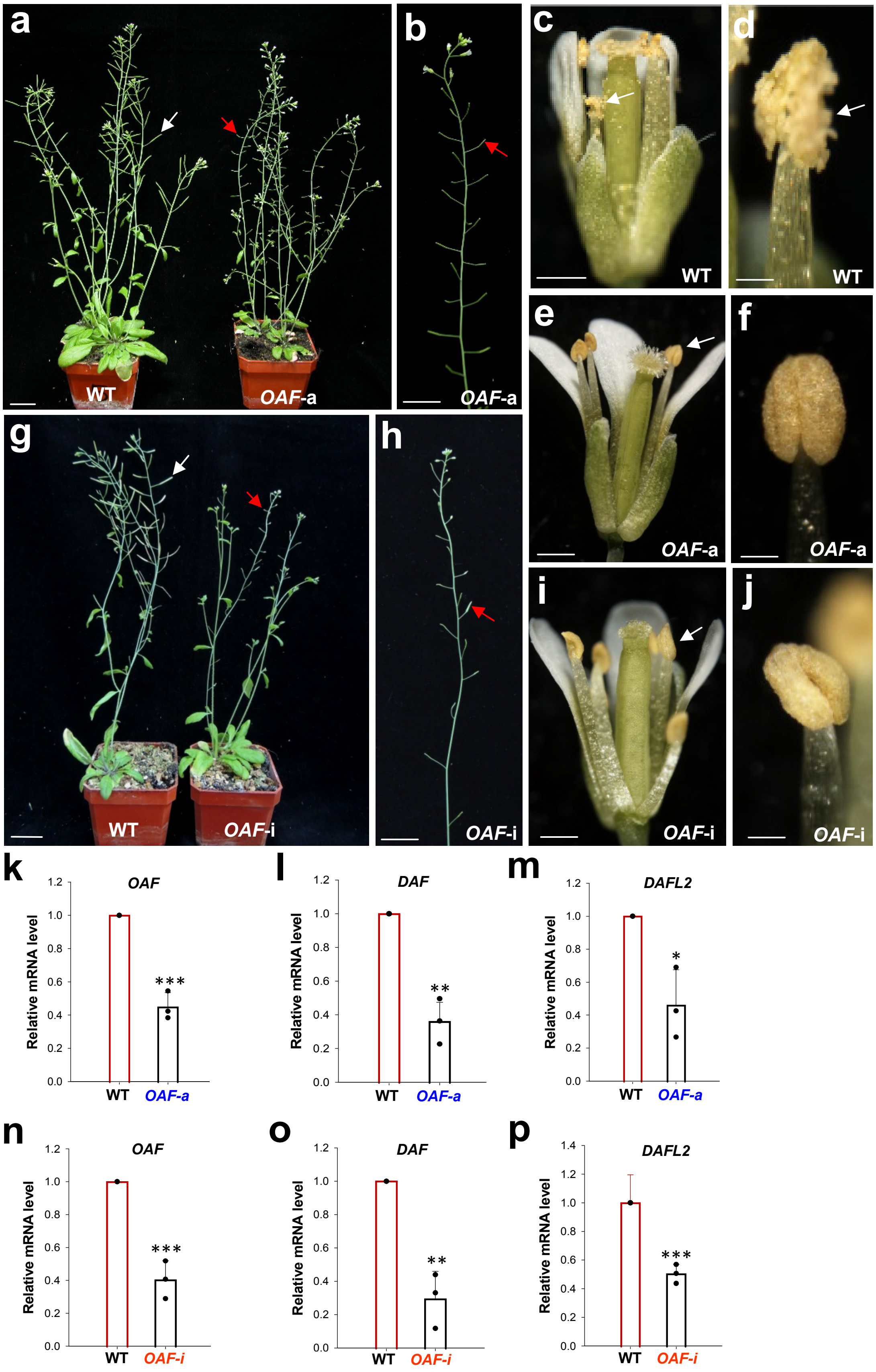

**Supplementary Fig. 4 The analysis of *OAF*-antisense and *OAF*-RNAi *Arabidopsis*.**

**a** An 35S::*OAF* antisense (*OAF*-a, right) produced short siliques without elongation (red arrow) whereas wild-type plants (WT, left) produced long, well-developed siliques (white arrow). Bar: 2 cm.

**b** Inflorescences from an 35S::*OAF* antisense (*OAF*-a) plant with short and un-elongated siliques (red arrow). Bar: 2 cm.

**c** In wild-type (WT) flowers, the anther was dehiscent and the pollen (arrowed) was released after stage 12. Bar: 0.5 mm

**d** Close-up of the dehiscent anther and the pollen (arrowed) from **(c)**. Bar: 0.1 mm.

**e** In 35S::*OAF* antisense (*OAF*-a) flowers, the anther was indehiscent (arrowed) even after stage 12. Bar: 0.5 mm.

**f** Close-up of the indehiscent anther from **(e)**. Bar: 0.1 mm.

**g** An 35S::*OAF* RNAi (*OAF*-i, right) produced short siliques without elongation (red arrow) whereas wild-type plants (WT, left) produced long, well-developed siliques (white arrow). Bar: 2 cm.

**h** Inflorescences from an 35S::*OAF* RNAi plant with short and un-elongated siliques (red arrow). Bar: 2 cm.

**i** In 35S::*OAF* RNAi (*OAF*-i) flowers, the anther was indehiscent (arrowed) even after stage 12. Bar: 0.5 mm.

**j** Close-up of the indehiscent anther from **(i)**. Bar: 0.1 mm.

**k-m** Analysis of the expression of *OAF* **(k)**, *DAF* **(l)** and *DAFL2* **(m)** in wild-type control (WT) and 35S::*OAF* antisense (*OAF*-a) plants. Error bars show ± SEM. n=3 biologically independent samples. The asterisks indicates significant difference from the wild-type (WT) value (*: $P \leq 0.05$, **: $P \leq 0.01$ and ***: $P \leq 0.001$). Statistical analysis was measured according to Student's t test.

**n-p** Analysis of the expression of *OAF* **(n),** *DAF* **(o)** and *DAFL2* **(p)** in wild-type control (WT) and 35S::*OAF* RNAi (*OAF*-i) plants. Error bars show ± SEM. n=3 biologically independent samples. The asterisks indicates significant difference from the wild-type (WT) value (**: $P \leq 0.01$ and ***: $P \leq 0.001$). Statistical analysis was measured according to Student's t test.

## Supplementary Fig. 5

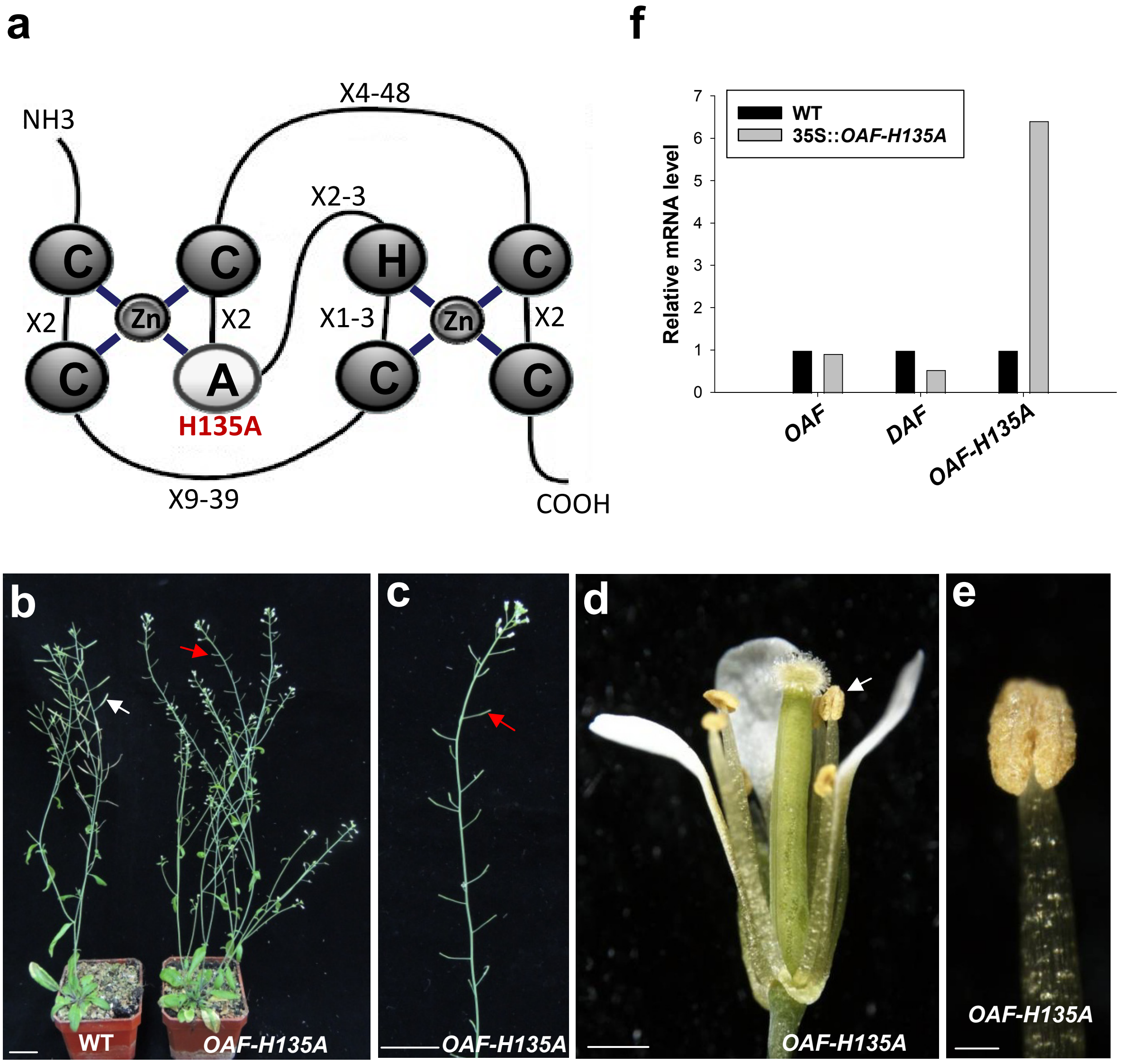

**Supplementary Fig. 5 The analysis of 35S::*OAF-H135A Arabidopsis*.**
**a** The positions of the eight conserved C3H2C3 residues in the RING finger motif of OAF are indicated. His-135 was substituted by Ala to generate *OAF-H135A*.
**b** An 35S::*OAF-H135A* (*OAF-H135A*, right) produced short siliques without elongation (red arrow) whereas wild-type plants (WT, left) produced long, well-developed siliques (white arrow). Bar: 2 cm.
**c** Inflorescences from an 35S::*OAF-H135A* plant with short and un-elongated siliques (red arrow). Bar: 2 cm.
**d** In 35S::*OAF-H135A* (*OAF-H135A*) flowers, the anther was indehiscent (arrowed) even after stage 12. Bar: 0.5 mm.
**e** Close-up of the indehiscent anther from **(d).** Bar: 0.1 mm.
**f** Analysis of the expression of endogenous *OAF, DAF* and transgenic *OAF-H135A* in wild-type control (WT) and 35S::*OAF-H135A* plant.

## Supplementary Fig. 6

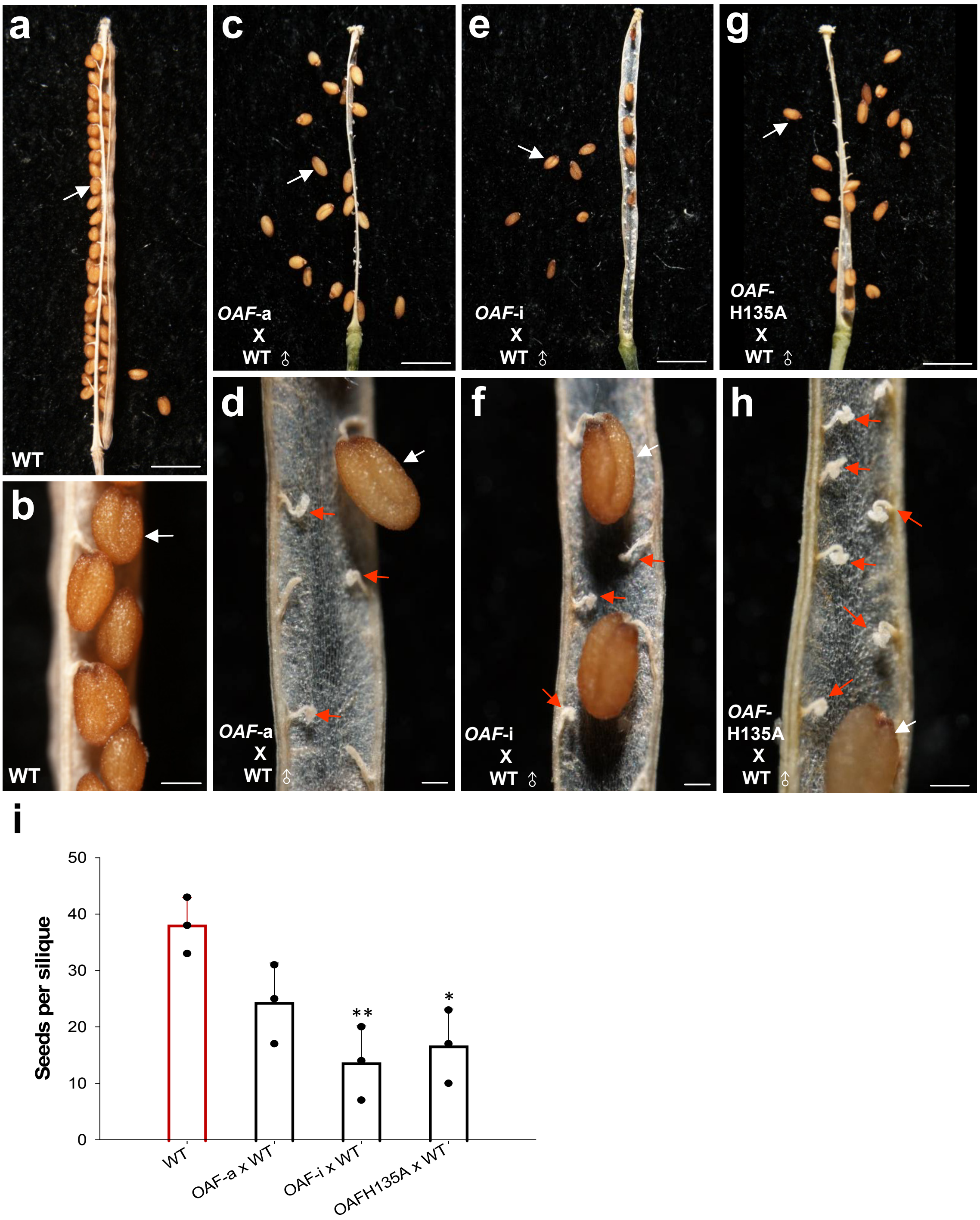

**Supplementary Fig. 6 The analysis of seed development in 35S::*OAF*-antisense x wild-type (WT) ♂, 35S::*OAF*-RNAi x wild-type (WT) ♂ and 35S::*OAF*-H135A x wild-type (WT) ♂ siliques.**

**a** Normally developing seeds (arrowed) were produced from a wild-type (WT) well-developed siliques. Bar: 1 mm.

**b** Close-up of the normally developed seeds (arrowed) from **(a)**. Bar: 0.2 mm.

**c** An 35S::*OAF*-antisense x wild-type (WT) ♂ partially elongated silique produced less than 20 seeds (arrowed). Bar: 1 mm.

**d** Defective and undeveloped seeds (red arrow) along with normal developing seeds (white arrow) were produced from an 35S::*OAF*-antisense x wild-type (WT) ♂ partially elongated silique. Bar: 0.1 mm.

**e** An 35S::*OAF*-RNAi x wild-type (WT) ♂ partially elongated silique produced less than 20 seeds (arrowed). Bar: 1 mm.

**f** Defective and undeveloped seeds (red arrow) along with normal developing seeds (white arrow) were produced from an 35S::*OAF*-RNAi x wild-type (WT) ♂ partially elongated silique. Bar: 0.1 mm.

**g** An 35S::*OAF*-H135A x wild-type (WT) ♂ partially elongated silique produced less than 20 seeds (arrowed). Bar: 1 mm.

**h** Defective and undeveloped seeds (red arrow) along with normal developing seeds (white arrow) were produced from an 35S::*OAF*-H135A x wild-type (WT) ♂ partially elongated silique. Bar: 0.1 mm.

**i** Comparison of total number of seeds produced in the 35S::*OAF*-antisense x wild-type (WT) ♂, 35S::*OAF*-RNAi x wild-type (WT) ♂, 35S::*OAF*-H135A x wild-type (WT) ♂ and wild-type (WT) siliques. Error bars show ± SEM. n=3 biologically independent samples. The asterisks "*" and "**" indicate significant differences from the wild-type (WT) value (*: $P \leq 0.05$ and **: $P \leq 0.01$). Statistical analysis was measured according to Student's t-test.

# Supplementary Fig. 7

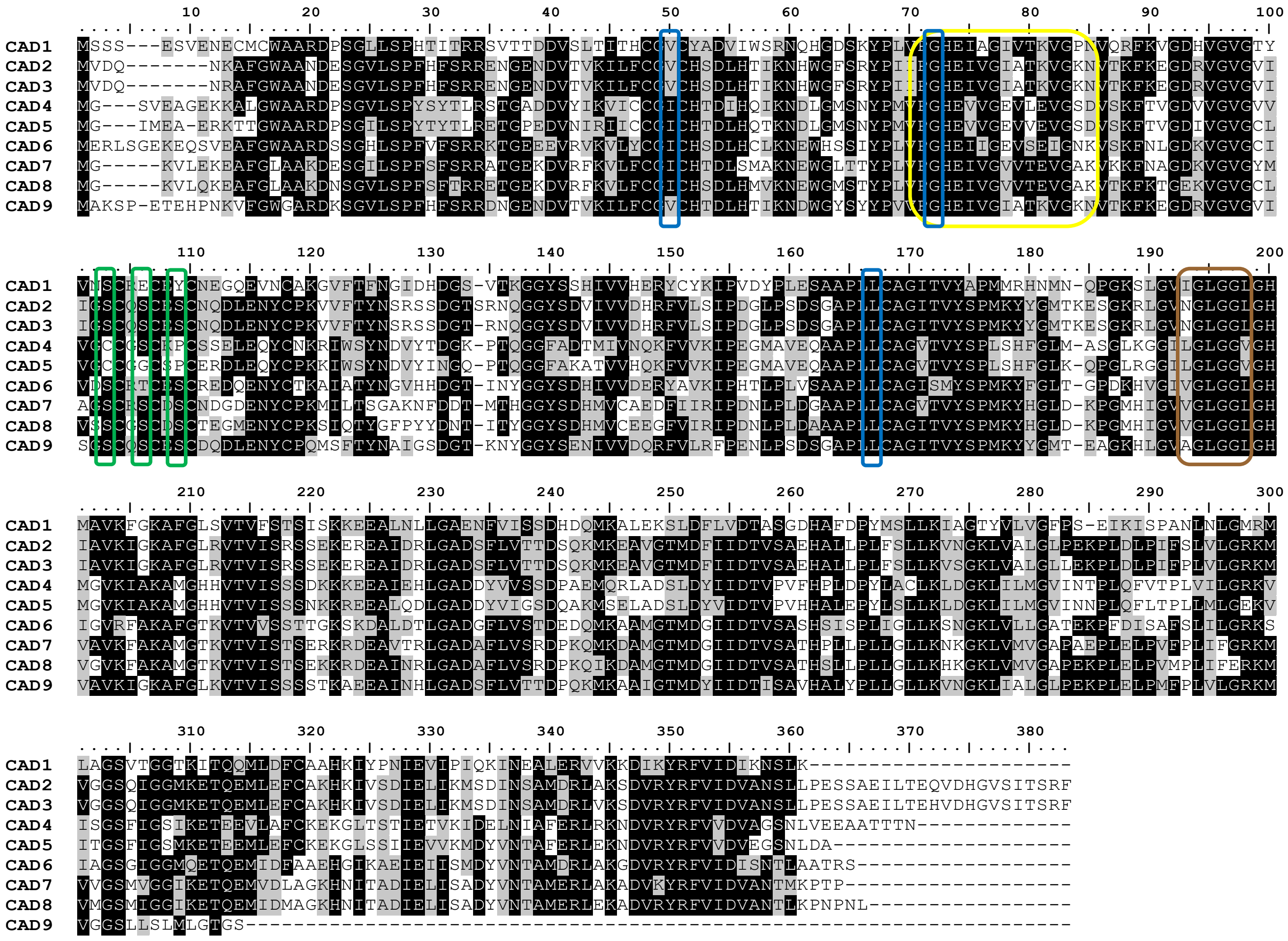

**Supplementary Fig. 7 Amino acid sequences alignment of nine *Arabidopsis* CAD-like proteins.**

Alignment of the amino acid sequences for *Arabidopsis* CAD1-9 proteins. The conserved Zn1 chelated site was boxed in blue, the conserved Zn2 chelated site was boxed in green, the conserved Zn-binding signature was boxed in yellow and the conserved NADPH-binding domain was boxed in brown. The dark highlights indicate identical residues and gray highlights indicate similar residues. Dashes were introduced to improve alignment. The amino acid sequences were aligned by the BioEdit program using ClustalW Multiple Alignment. This sequence alignment was generated by the ClustalW-Multiple Sequence Alignment Program at the DNA Data Bank of Japan (http://clustalw.ddbj.nig.ac.jp/top-e.html).

# Supplementary Fig. 8

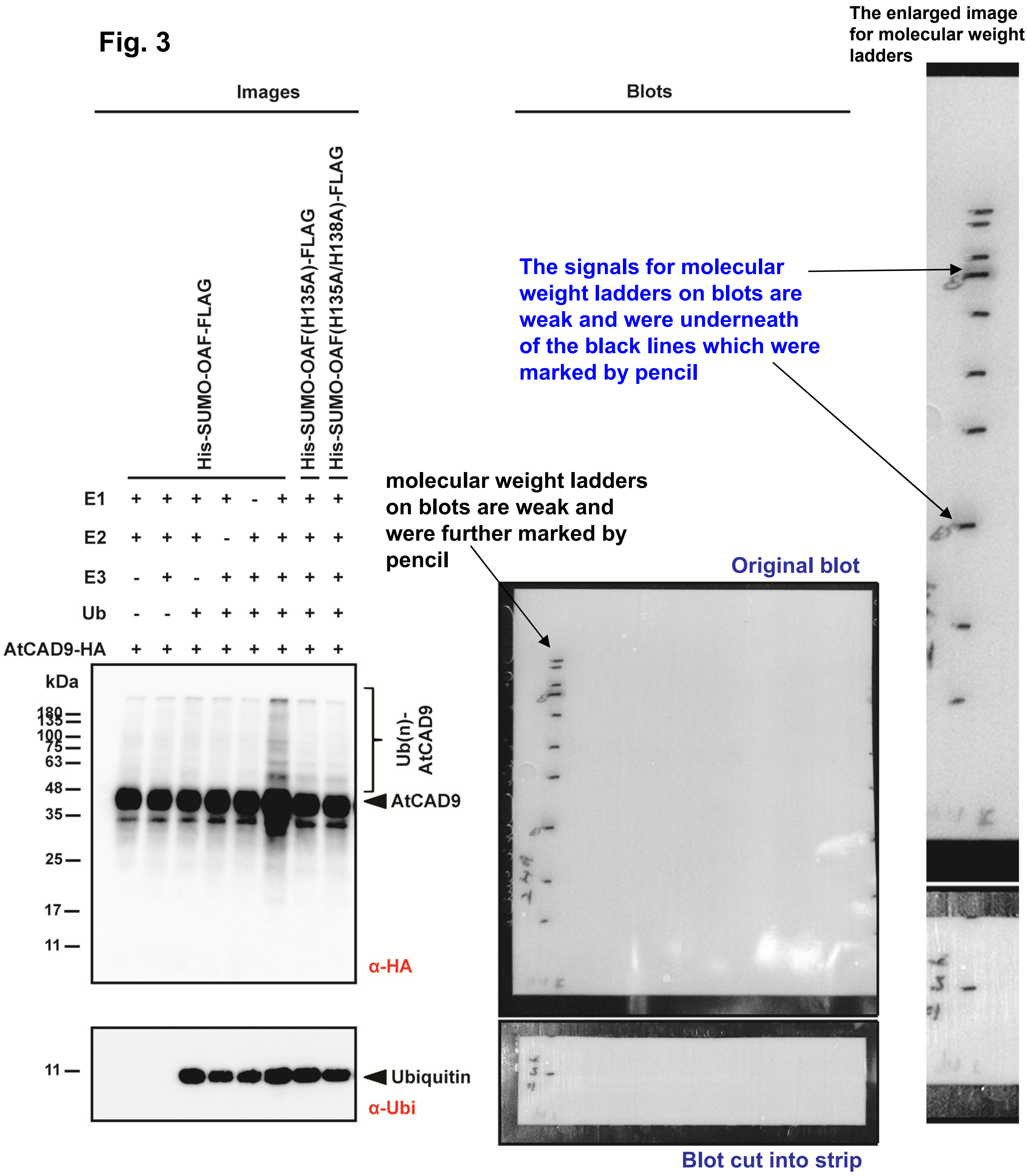

**Supplementary Fig. 8 The original blots used for Western blot analysis in Figure 3.**

1. After ubiquitination reaction, proteins and molecular weight markers were separated together by SDS-PAGE and transferred into PVDF membranes.
2. The signals for molecular weight ladders on blots are weak (indicated by arrows) and were underneath of the black lines which were marked by pencil in order to capture clear images.
3. In Figure 3, two gels were performed and **two blots** were generated.
   (1) One of the blots was hybridized with anti-HA antibody to detect the AtCAD9 and high molecular weight ubiquitinated-AtCAD9 (upper panel, Figure 3).
   (2) Another blot was cut into strip and the strip with low molecular weight proteins was hybridized with anti-ubiquitin antibody to only detect low molecular weight ubiquitin (lower panel, Figure 3).
4. Chemiluminescence signals generated by antibodies using ECL reagents (PerkinElmer) were captured with the ImageQuant LAS 4000 mini (GE Healthcare) (upper- and lower-left panels in the Supp Figure 8). At the same time, the blot images were captured under white light (upper- and lower-right panels in the Supp Figure 8).

# Supplementary Fig. 9

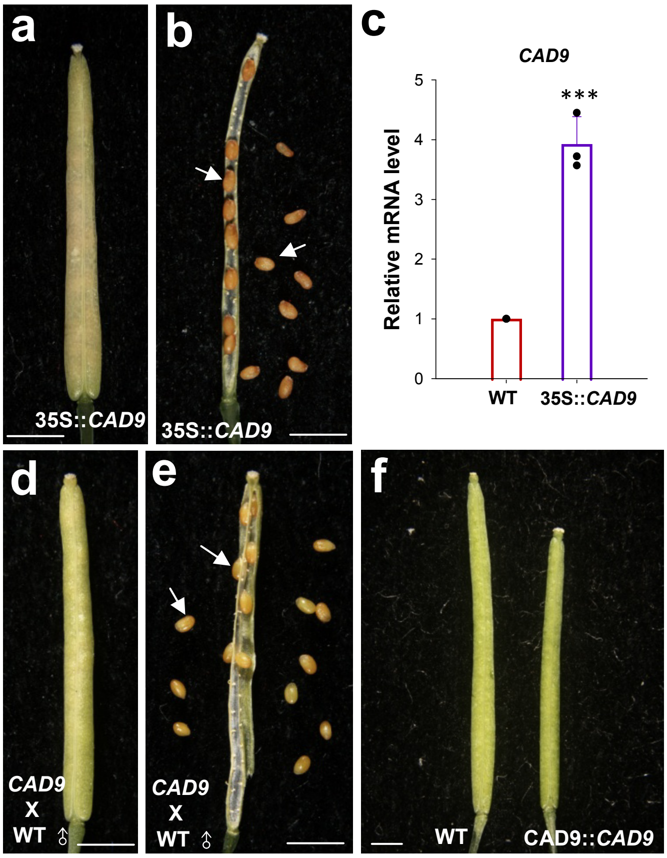


**Supplementary Fig. 9 The analysis of seed development in 35S::*CAD9* and 35S::*CAD9* x wild-type (WT) ♂ siliques.**

**a** An 35S::*CAD9* partially elongated silique. Bar: 1 mm.

**b** An 35S::*CAD9* partially elongated silique produced less than 20 seeds (arrowed). Bar: 1 mm.

**c** Analysis of the expression of *CAD9* in wild-type control (WT) and 35S::*CAD9* plants. Error bars show ± SEM. n=3 biologically independent samples. The asterisks indicates significant difference from the wild-type (WT) value (***: $P \leq 0.001$). Statistical analysis was measured according to Student's t test.

**d** An 35S::*CAD9* x wild-type (WT) ♂ partially elongated silique. Bar: 1 mm.

**e** An 35S::*CAD9* x wild-type (WT) ♂ partially elongated silique produced less than 20 seeds (arrowed). Bar: 1 mm.

**f** An CAD9::*CAD9* partially elongated silique (right) along with a wild-type (WT) well-developed silique (left). Bar: 1 mm.

Supplementary Fig. 10

**a**

```
   1 AAAAAGTGACCATTCACTTGCTACATATATAAATAATAACAATGATATGG

  51 TTTTTTTAAAACCAAAGAATTTCTTGGACCAACCACATCATCATATCTCT

 101 CAAAAGAGAGTCGCCCATAAATTCATTTATGACTCCTTTTATCACAATCT

 151 TGCCATAAATATTTTTGCCTCCTCCCATCTTTACACACAAGTCACGAGGA

 201 AGAAGCAAATCAGAAAAAGATCAAAGCTCAGAAATAAAGAGTACAGAGAT
                                                uOAF
 251 TAATTCATTAAGAGTTTTTAAGAACATGAGGGATATCGATTCTCTCTTCT
                               M  R  D  I  D  S  L  F
 301 CCATCTCTTTTGTCATTCGATTTGTCCAACAAGACGAGTCTTGAAGGTGA
     S  I  S  F  V  I  R  F  V  Q  Q  D  E  S  *
 351 AGTGATCAAGCTTTTTTTTTCCTTCTCTCCTCTGCTTCTCAAGCGTACCT
                                ath-miR847
 401 CAAAAGATGGGTCGGTTGCTACTTGAGCCGCAAGCAAACGCACCCGCCAA
           M  G  R  L  L  L  E  P  Q  A  N  A  P  A  N
 451 CGCAAACCCTAAACCCAAAGGAGGCATAAACGACACCTACTTCGACACTA
       A  N  P  K  P  K  G  G  I  N  D  T  Y  F  D  T
 501 ACATGGTTATCATTTTAGCTGCTTTACTCTGCGCTTTAATCTGTGCTCTA
     N  M  V  I  I  L  A  A  L  L  C  A  L  I  C  A  L
 551 AGCCTCAACTCTGCCTTGCGATGTGTGCTACGCATAACTCGGAGATTCAC
      S  L  N  S  A  L  R  C  V  L  R  I  T  R  R  F  T
 601 TTCGGATGATCAAGTCTCAAACGCTTCAAACGCAAACGCAAATTTGGGAC
       S  D  D  Q  V  S  N  A  S  N  A  N  A  N  L  G
 651 GTTTAGCGGCTGCCACGGGTCTCAAGAAACAGGCGTTGAAACAAATCCCC
     R  L  A  A  A  T  G  L  K  K  Q  A  L  K  Q  I  P
 701 GTGGGACTGTACGGATCAGGAATCATAGACATGAAAGCTACGGAGTGTTT
      V  G  L  Y  G  S  G  I  I  D  M  K  A  T  E  C  L
 751 GATCTGTCTGGGAGATTTCGAAGATGGAGAAAAAGTTAGGGTTTTACCTA
       I  C  L  G  D  F  E  D  G  E  K  V  R  V  L  P
 801 AATGTAACCATGGCTTCCACGTGAGGTGCATCGATACTTGGTTGCTCTCA
     K  C  N  H  G  F  H  V  R  C  I  D  T  W  L  L  S
 851 CGTTCCTCTTGCCCCACTTGTAGACAATCGCTACTCCTTGAGCAGCCTTC
      R  S  S  C  P  T  C  R  Q  S  L  L  L  E  Q  P  S
 901 GCCGATGGCTGTTTCCCGGCGGGACGAGGACATGGTAGTTTCCATCGTAT
       P  M  A  V  S  R  R  D  E  D  M  V  V  S  I  V
 951 AGGGACATGATCGGTGATTTCTTAATTATGCTCTCTCAAGTGGGCTTGGT
     *
1001 TTTTGTTGGTTGGAATTGTTTGTGTGTGTGCAAACTAATGTGATTTATTT

1051 TCACATATTCTTGTTCTGATGAATTTAATTAGTGTGATGGGTTTTCTTGA

1101 AATCAAGCA
```

**b**

```
      AtOAF   99  UCUCUCCUCUGCUUCU  114
                  || ||||||| |||||
ath-miR847     1  UCACUCCUCUUCUUCU   16
```

**Supplementary Fig. 10 Sequence analysis for 5'-, 3'-UTR and ORF regions for *OAF* gene.**
**a** Based on the sequence analysis, an uORF (upstream open reading frame) named *uOAF* which contains 69 bp and encodes 22 amino acids (in green color), was found in the upstream 5'UTR of *OAF* genes. A putative 16 bp target sequences for miR847 (TCTCTCCTCTGCTTCT) (in red color) was identified in 5'UTR between *uOAF* and *OAF* coding region (in purple color). Amino acid sequences for OAF protein were listed under the DNA sequences.
**b** Sequence alignment for the 16 bp target sequences for miR847 (ath-miR847) and for *OAF* 5'UTR region (AtOAF). The mismatches between two sequences were in red color.

## Supplementary Fig. 11

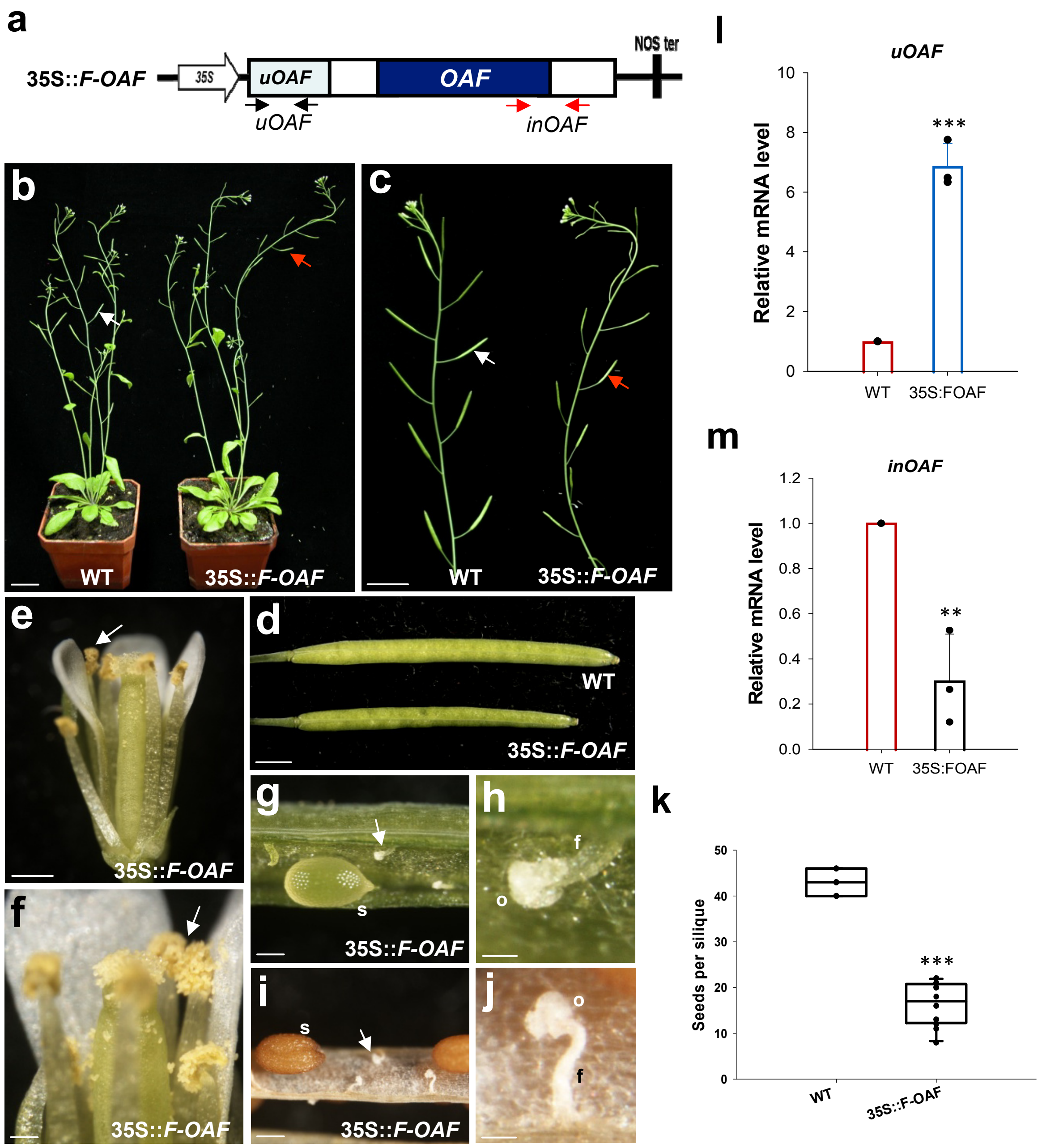

**Supplementary Fig. 11 The analysis of 35S::*F-OAF Arabidopsis*.**
**a** A diagram of the 35S::*F-OAF* construct which contains gene structure (dark blue box) for *OAF* and its regulatory regions, uOAF (light blue box) in 5'-UTR, 5'-UTR and 3'-UTR regions (white boxes). The two red arrows are the primer pair (*inOAF*) used for detection of the internal *OAF* gene expression. The two black arrows are the primer pair (*uOAF*) used for detection of the *uOAF* expression.
**b** An 35S::*F-OAF* plant (right) produced partially elongated siliques (red arrow) whereas wild-type plants (WT, left) produced long, well-developed siliques (white arrow). Bar: 2 cm.
**c** Inflorescences from an 35S::*F-OAF* plant (right) with partially elongated siliques (red arrow) and inflorescence with elongated siliques (white arrow) from a wild-type plant (WT, left). Bar: 1 cm.
**d** Close-up of the siliques from 35S::*F-OAF* (bottom) and wild-type plant (WT, top). Bar: 1 mm.
**e** In 35S::*F-OAF* flowers, the anther was dehiscent and the pollen (arrowed) was released after stage 12. Bar: 0.5 mm.
**f** Close-up of the dehiscent anther and the pollen (arrowed) from **(e)**. Bar: 0.1mm.
**g** Defective and undeveloped ovules (arrowed) along with normal developing seeds (s) were produced from an 35S::*F-OAF* partially elongated silique. Bar: 0.1 mm.
**h** Close-up of the defective and undeveloped ovules (o) from **(g)**. f: funiculus. Bar: 0.03 mm.
**i** Defective and undeveloped ovules (arrowed) along with normally developed seeds (s) observed from a mature 35S::*F-OAF* partially elongated silique. Bar: 0.1 mm.
**j** Close-up of the defective and undeveloped ovules (o) from **(i)**. f: funiculus. Bar: 0.03 mm.
**k** Comparison of total number of seeds produced in the 35S::*F-OAF* and wild-type (WT) siliques. Error bars show ± SEM. n=10 and 20 biologically independent samples for wild-type and 35S::*F-OAF*, respectively. The asterisks "***" indicate significant differences from the wild-type (WT) value (***: $P \leq 0.001$). Statistical analysis was measured according to Student's t-test. Box plots showed the median, 10th, 25th, 75th and 90th percentiles as vertical boxes with error bars.
**l-m** Analysis of the expression of u*OAF* **(l)** and inOAF **(m)** in wild-type control (WT) and 35S::*F-OAF* plants. Error bars show ± SEM. n=3 biologically independent samples. The asterisks "**" and "***" indicate significant differences from the wild-type (WT) value (**: $P \leq 0.01$ and ***: $P \leq 0.001$). Statistical analysis was measured according to Student's t-test.

## Supplementary Fig. 12

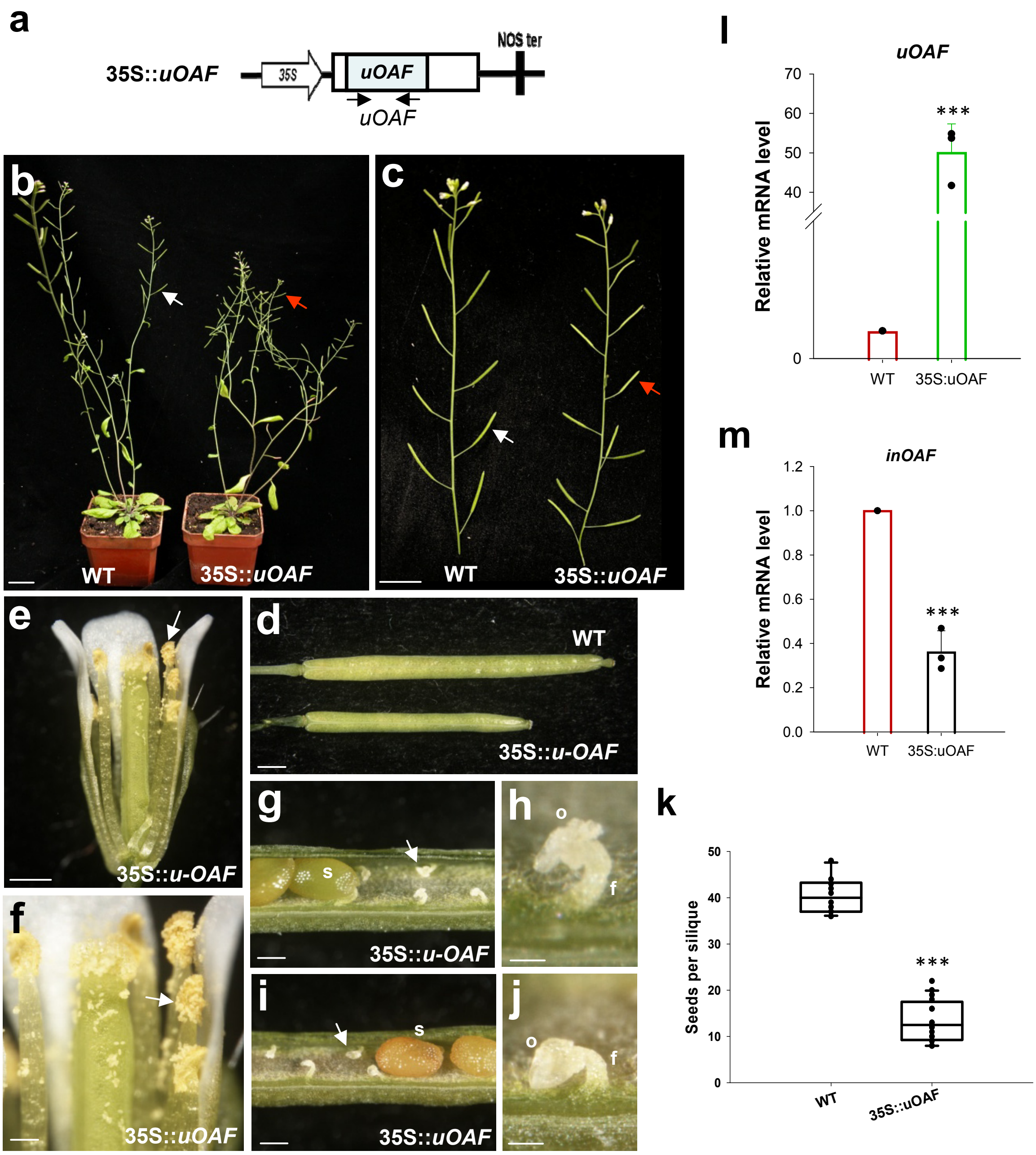

**Supplementary Fig. 12 The analysis of 35S::*uOAF Arabidopsis*.**
**a** A diagram of the 35S::*uOAF* construct which contains uOAF (light blue box) in 5'-UTR regions (white boxes) of *OAF* gene. The two arrows are the primer pair (*uOAF*) used for detection of the *uOAF* expression.
**b** An 35S::*uOAF* plant (right) produced partially elongated siliques (red arrow) whereas wild-type plants (WT, left) produced long, well-developed siliques (white arrow). Bar: 2 cm.
**c** Inflorescences from an 35S::*uOAF* plant (right) with partially elongated siliques (red arrow) and inflorescence with elongated siliques (white arrow) from a wild-type plant (WT, left). Bar: 1 cm.
**d** Close-up of the siliques from 35S::*uOAF* (bottom) and wild-type plant (WT, top). Bar: 0.1 cm.
**e** In 35S::*uOAF* flowers, the anther was dehiscent and the pollen (arrowed) was released after stage 12. Bar: 0.5 mm.
**f** Close-up of the dehiscent anther and the pollen (arrowed) from **(e)**. Bar: 0.1 mm.
**g** Defective and undeveloped ovules (arrowed) along with normal developing seeds (s) were produced from an 35S::*uOAF* partially elongated silique. Bar: 0.1 mm.
**h** Close-up of the defective and undeveloped ovules (o) from **(g)**. f: funiculus. Bar: 0.03 mm.
**i** Defective and undeveloped ovules (arrowed) along with normally developed seeds (s) observed from a mature 35S::*uOAF* partially elongated silique. Bar: 0.1 mm.
**j** Close-up of the defective and undeveloped ovules (o) from **(i)**. f: funiculus. Bar: 0.03 mm.
**k** Comparison of total number of seeds produced in the 35S::*uOAF* and wild-type (WT) siliques. Error bars show ± SEM. n=10 and 20 biologically independent samples for wild-type and 35S::*uOAF*, respectively. The asterisks "***" indicate significant differences from the wild-type (WT) value (***: $P \leq 0.001$). Statistical analysis was measured according to Student's t-test. Box plots showed the median, 10th, 25th, 75th and 90th percentiles as vertical boxes with error bars.
**l-m** Analysis of the expression of u*OAF* **(l)** and in*OAF* **(m)** in wild-type control (WT) and 35S::*uOAF* plants. Error bars show ± SEM. n=3 biologically independent samples. The asterisks "***" indicate significant differences from the wild-type (WT) value (***: $P \leq 0.001$). Statistical analysis was measured according to Student's t-test.

## Supplementary Fig. 13

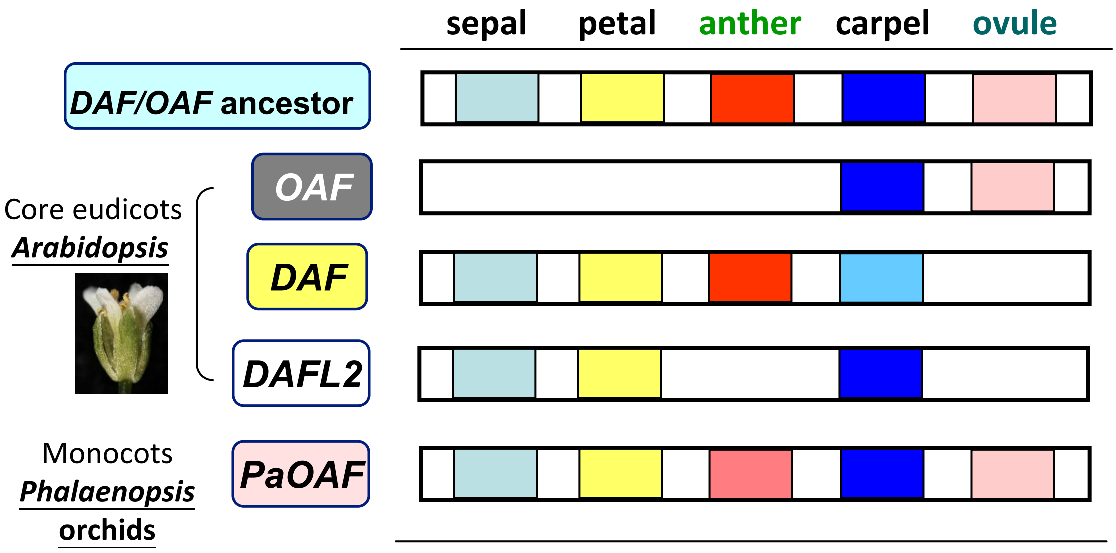


**Supplementary Fig. 13 The expression divergence and distinct function model for duplicated *DAF/OAF*-like genes in plants.**

In plants, the *DAF/OAF*-like ancestor may have the functions in regulating sepal/petal/anther/carpel/ovule development. In the eudicot *Arabidopsis*, *DAF/OAF* ancestor duplicated into three genes (*DAF, OAF, DAFL1*) through two duplication events and further evolved to divergent functions through subfunctionalization to maintain partial function for ancestor, which may overlapped to each other or unique for one gene, in regulating various flower development. In this case, *OAF* regulates ovule/carpel development, *DAF* regulates sepal/petal/anther/carpel development whereas *DAFL2* regulates sepal/petal/carpel development. In the monocots *Phalaenopsis* orchid (*Orchidaceae*), only one *PaOAF* gene remained from *DAF/OAF* ancestor which is likely evolved through nonfunctionalization. *PaOAF* maintained the conserved functions as ancestor in regulating sepal/petal/anther/carpel/ovule development. However, the function in regulating anther dehiscence was absent for *PaOAF* due to the production of a unique anther cap and a pollinium structure in orchid during evolution. The putative functions for each gene in regulating sepal/petal/anther/carpel/ovule development were indicated in boxes with different colors.

## Supplementary Fig. 14

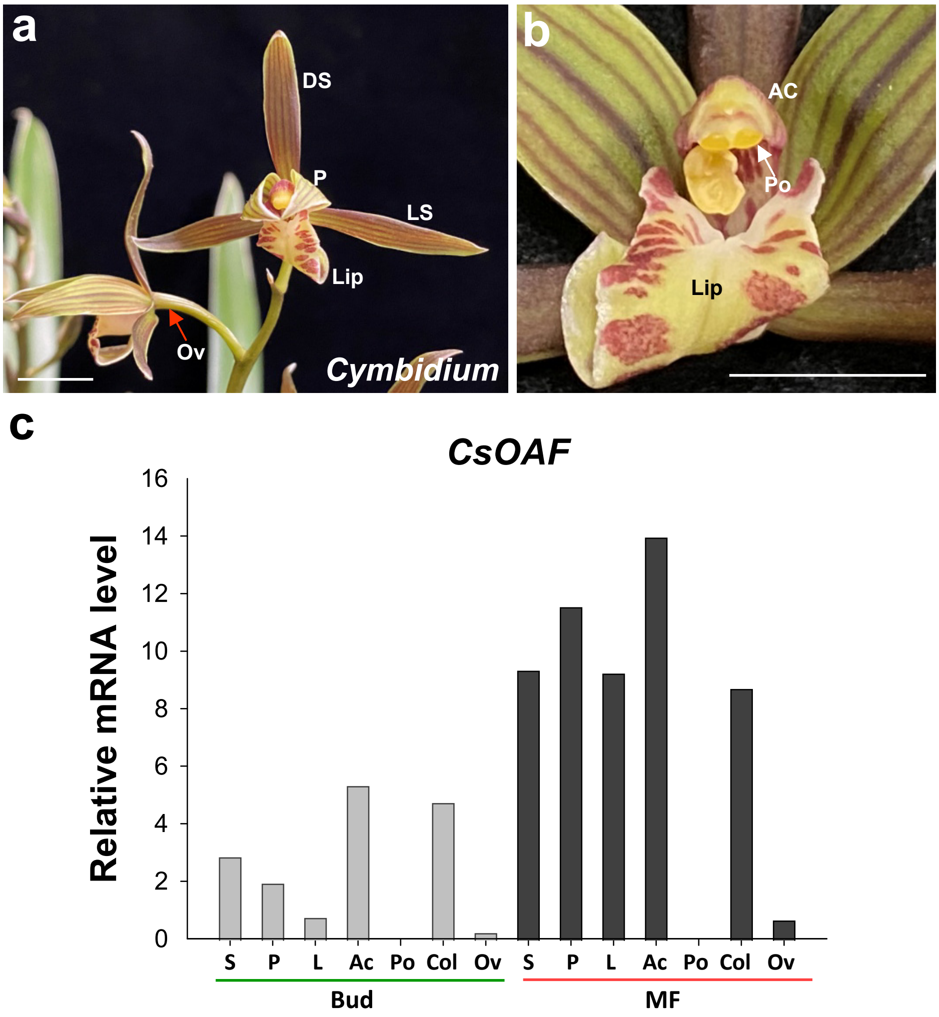


**Supplementary Fig. 14 The analysis of *CsOAF* expression in *Cymbidium*.**
**a** A flower of wild-type *Cymbidium* spp. The ovary (Ov) was indicated by red arrow. Lip: lips, P: petals, DS: dorsal sepals, LS: lateral sepals. Bar: 1 cm.
**b** Close-up of the anther cap (Ac) and pollinia (Po) of the *Cymbidium* spp flower. Lip: lips. Bar: 1 cm.
**c** Analysis of the expression of *CsOAF* in sepal (S), petal (P), lips (L), anther cap (Ac), pollinia (Po), column (Col) and ovary (Ov) of the *Cymbidium* spp flower bud (Bud) and mature flowers (MF).

## Supplementary Fig. 15

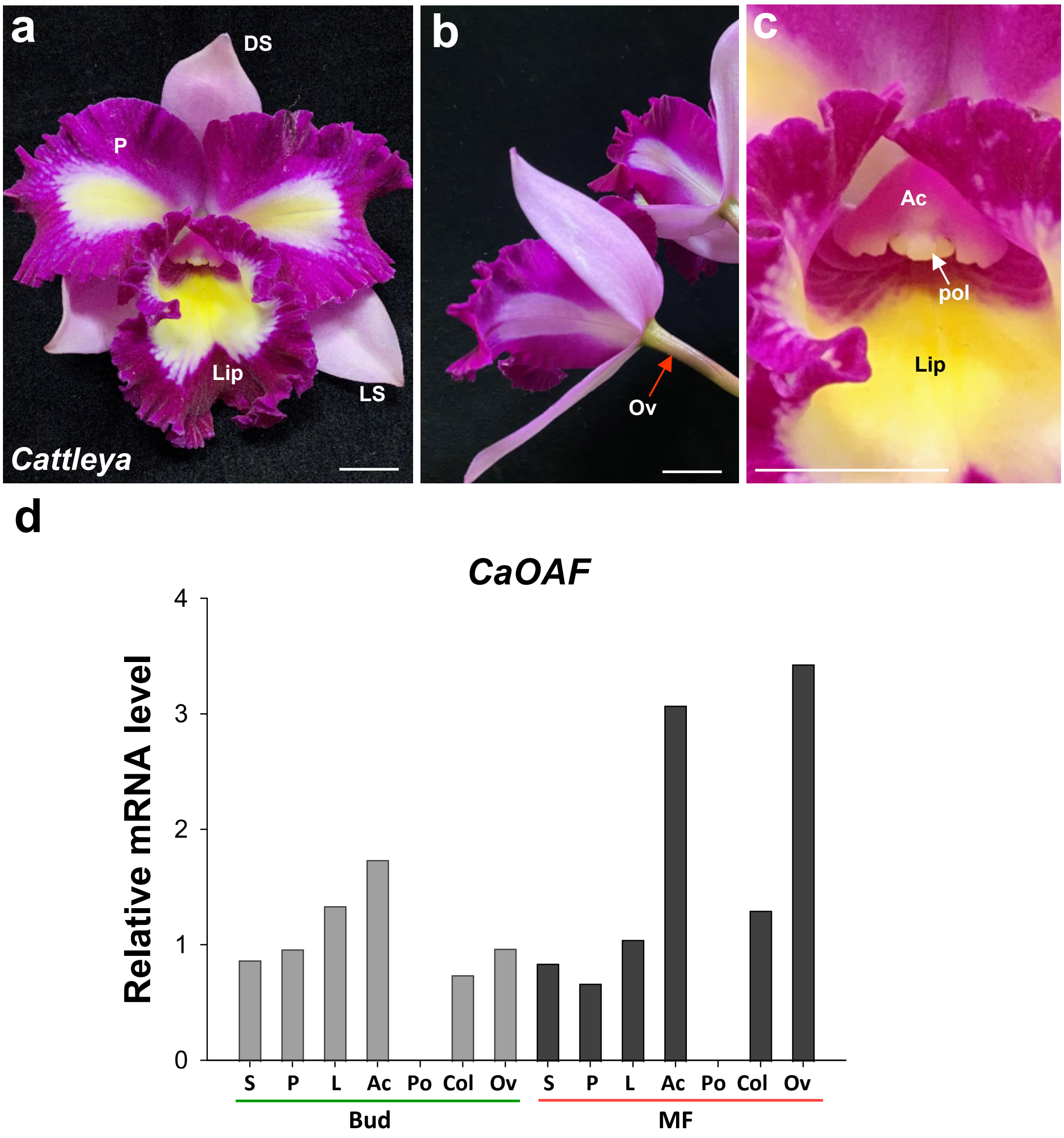


**Supplementary Fig. 15 The analysis of *CaOAF* expression in *Cattleya.***

**a** A flower of wild-type *Cattleya* spp. Lip: lips, P: petals, DS: dorsal sepals, LS: lateral sepals. Bar: 1 cm.

**b** The back side of the *Cattleya* spp. flowers revealed the organ of the ovary (Ov). Bar: 1 cm.

**c** Close-up of the anther cap (Ac) and pollinia (Po) of the *Cattleya* spp. flower. Lip: lips. Bar: 1 cm.

**d** Analysis of the expression of *CaOAF* in sepal (S), petal (P), lips (L), anther cap (Ac), pollinia (Po), column (Col) and ovary (Ov) of the *Cattleya* spp flower bud (Bud) and mature flowers (MF).

**Supplementary Table 1**. The putative interacting proteins of OAF identified by a yeast two-hybrid (YTH) analysis.

| Gene Symbol | Repeats[1] | Description | Function | Major expression |
|---|---|---|---|---|
| **AT4G39330** | **4** | **CINNAMYL ALCOHOL DEHYDROGENASE 9 (CAD9)** | **lignin biosynthetic process** | **carpel** |
| AT4G13930 | 5 | SERINE HYDROXYMETHYLTRANSFERASE 4 | folic acid metabolic process | stamen |
| AT3G62250 | 2 | UBIQUITIN 5 | protein ubiquitination | seed |
| AT3G52590 | 2 | UBIQUITIN EXTENSION PROTEIN 1 | protein ubiquitination | seed |
| AT2G17200 | 2 | DOMINANT SUPPRESSOR OF KAR 2 | ubiquitin-dependent protein catabolic process | senescing leaf |
| AT5G42220 | 2 | Ubiquitin-like superfamily protein | ubiquitin-dependent ERAD pathway | pollen |
| AT5G53300 | 1 | UBIQUITIN-CONJUGATING ENZYME 10 | protein polyubiquitination | pollen |
| AT2G36170 | 1 | UBIQUITIN EXTENSION PROTEIN 2 | protein ubiquitination | shoot apex meristem |
| AT5G66570 | 1 | PS II OXYGEN-EVOLVING COMPLEX 1 | photosystem II stabilization | leaf |
| AT5G01600 | 1 | ARABIDOPSIS THALIANA FERRETIN 1 | photoxidative stress | sepal |
| AT3G05430 | 1 | PWWP DOMAIN PROTEIN 4 | DNA-templated transcription | carpel |
| AT1G08970 | 1 | NUCLEAR FACTOR Y, SUBUNIT C9 | seed germination | seed |
| AT2G27710 | 1 | RIBOSOMAL PROTEIN P2Y | cold stress response | shoot apex meristem |
| AT1G30380 | 1 | PHOTOSYSTEM I SUBUNIT K | chlorophyll biosynthetic process | leaf |
| AT1G03600 | 1 | PHOTOSYSTEM II FAMILY PROTEIN 27 | photosystem II assembly | leaf |
| AT3G50820 | 1 | PHOTOSYSTEM II SUBUNIT O-2 | photosystem II assembly | leaf |
| AT1G08980 | 1 | AMIDASE 1 | indoleacetic acid biosynthetic process | leaf |
| AT5G50920 | 1 | CLPC HOMOLOGUE 1 | chloroplast organization | sepal |
| AT4G05320 | 1 | POLYUBIQUITIN 10 | protein ubiquitination | seed |
| AT1G29150 | 1 | NON-ATPASE SUBUNIT 9 | ubiquitin-dependent protein catabolic process | seed |
| AT3G52920 | 1 | transcriptional activator (DUF662) | RNA metabolic process | seed |
| AT5G42220 | 1 | Ubiquitin-like superfamily protein | ubiquitin-dependent ERAD pathway | pollen |
| AT3G55605 | 1 | Mitochondrial glycoprotein family protein | - | shoot apex meristem |
| AT1G29880 | 1 | glycyl-tRNA synthetase / glycine-tRNA ligase | mitochondrial glycyl-tRNA aminoacylation | leaf |
| AT5G65180 | 1 | ENTH/VHS family protein | mRNA 3'-end processing | seed |

[1:] Indicated the number of positive clones containing the sequence for this gene.

**Supplementary Table 2**. Oligo nucleotide sequence of primers used in gene cloning.

| Primer name | Primer sequence | Restriction site |
|---|---|---|
| **proOAF-F** | 5'- TCTAGACTTTAATGCCCATATGCTGAG -3' | XbaI |
| **proOAF-R** | 5'- GGATCCTCACCTTCAAGACTCGTC -3' | BamHI |
| **proCAD9-F** | 5'- AAGCTTGAGGCGAAAACAGAGTAGGTG -3' | HindIII |
| **proCAD9-R** | 5'- CTGCAGAAAGACTTTGTTCGGATGCTC -3' | PstI |
| **proCAD9-AtCAD9-R** | 5'- GTCGACTGGAGGGCTCAAGGAGTTAGC -3' | SalI |
| **OAF-F** | 5'- GGTACCATGGGTCGGTTGCTACTTG -3' | KpnI |
| **OAF-R** | 5'- GGTACCCTATACGATGGAAACTACCATGT -3' | KpnI |
| **OAF-RNAi-F1** | 5'- TCTAGAGCTTCAAACGCAAACGC -3' | XbaI |
| **OAF-RNAi-R1** | 5'- CTGCAGCATGTCCTCGTCCCGCC -3' | PstI |
| **OAF-RNAi-F2** | 5'- CTCGAGGCTTCAAACGCAAACGC -3' | XhoI |
| **OAF-RNAi-R2** | 5'- AAGCTTCATGTCCTCGTCCCGCC -3' | HindIII |
| **OAFH135A-F** | 5'- GTAACGCTGGCTTCCACGTG -3' | |
| **OAFH135A-R** | 5'- AGCCAGCGTTACATTTAGGTAAAACC -3' | |
| **CAD9-F** | 5'- CTGCAGATGGCGAAATCTCCAGAAACAGAG -3' | PstI |
| **CAD9-R** | 5'- GTCGACTCATGGAGGGCTCAAGGAGTTAG -3' | SalI |
| **FOAF-F** | 5'- CTGCAGACATGAGGGATATCGATTCTCTC -3' | PstI |
| **FOAF-R** | 5'- GTCGACCTATACGATGGAAACTACCATG -3' | SalI |
| **uOAF-F** | 5'- CTGCAGGCCATAAATATTTTTGCCTCCTCC -3' | PstI |
| **uOAF-R** | 5'- GTCGACCTTTTGAGGTACGCTTGAGAAG -3' | SalI |
| **miR847-F** | 5'- CTGCAGCATTTTCTTGTATTCAAAGTATCTTAA GTTAAGAAG -3' | PstI |
| **miR847-R** | 5'-GTCGACATATCTAACAGATTTTTCACTCCATCT AA -3' | SalI |
| **VIGS-PaOAF-F** | 5'- **GGGGACAAGTTTGTACAAAAAAGCAGGCT** CTCCGTCCTCGATAGTTCTGTG -3' | Sequences in bold letters represent recombination sites |
| **VIGS-PaOAF-R** | 5'- **GGGGACCACTTTGTACAAGAAAGCTGGGT** TCAGCTCATCTCCACCAACTC -3' | Sequences in bold letters represent recombination sites |

**Supplementary Table 3**. Oligo nucleotide sequence of primers used in PCR analysis.

| **Gene name** | **Primer name** | **Primer sequence** |
|---|---|---|
| ***UBQ10*** | RT-UBQ10-F | 5'- CTCAGGCTCCGTGGTGGTATG -3' |
| | RT-UBQ10-4-2 | 5'- GTGATAGTTTTCCCAGTCAACGTC -3' |
| ***OAF*** | RT-AtOAF-F | 5'- ACTGTACGGATCAGGAATCATAGAC -3' |
| | RT-AtOAF-R | 5'- CCACTTGAGAGAGCATAATTAAGAAATC -3' |
| ***internal OAF*** | RT-AtinOAF-F | 5'- AGTTTCCATCGTATAGGG -3' |
| | RT-AtinOAF-R | 5'- AACAATTCCAACCAACAA -3' |
| ***upstream OAF*** | RT-AtuOAF-F | 5'- ACATGAGGGATATCGATTCTCTC -3' |
| | RT-AtuOAF-R | 5'- CTTCAAGACTCGTCTTGTTGGAC -3' |
| ***CAD9*** | RT-AtCAD9-F | 5'- GAGGCAGGGAAGCATTTAGGG -3' |
| | RT-AtCAD9-R | 5'- ACAAGAAACGAATCAGCACCAAG -3' |
| ***PaACT4*** | PACT4-RT-F | 5'- CTTACTGAGGCACCGCTGAAC -3' |
| | PACT4-RT-R | 5'- CCATCACCAGAATCAAGAACAATACC -3' |
| ***PaOAF*** | RT-PaOAF-F | 5'- GGAGTTGGTGGAGATGAG -3' |
| | RT-PaOAF-R | 5'- TCTACTGCGTGAGATATTCG -3' |
| ***OnACT*** | OnAT-RT-3 | 5'- GGATTAGGCTCTCTGCTGTTGG -3' |
| | OnAT-RT-4 | 5'- GTGTGGATAAGACGCTGTTGTATG -3' |
| ***CsOAF*** | RT-CsOAF-F | 5'- ACGCAGGAGATGATAGAT -3' |
| | RT-CsOAF-R | 5'- GTTCACATAATCGGCTTTG -3' |
| ***CaOAF*** | RT-CaOAF-F | 5'- AATATGCGCGCTAGGACTG -3' |
| | RT-CaOAF-R | 5'- CGTTGCCTCCGTAGTCTC -3' |
